# Interspecific protection against oxidative stress: green algae protect harmful cyanobacteria against hydrogen peroxide

**DOI:** 10.1111/1462-2920.15429

**Published:** 2021-02-21

**Authors:** Erik F. J. Weenink, Hans C. P. Matthijs, J. Merijn Schuurmans, Tim Piel, Maria J. van Herk, Corrien A. M. Sigon, Petra M. Visser, Jef Huisman

**Affiliations:** ^1^ Department of Freshwater and Marine Ecology, Institute for Biodiversity and Ecosystem Dynamics University of Amsterdam P.O. Box 94240 Amsterdam GE 1090 The Netherlands

## Abstract

Oceanographic studies have shown that heterotrophic bacteria can protect marine cyanobacteria against oxidative stress caused by hydrogen peroxide (H_2_O_2_). Could a similar interspecific protection play a role in freshwater ecosystems? In a series of laboratory experiments and two lake treatments, we demonstrate that freshwater cyanobacteria are sensitive to H_2_O_2_ but can be protected by less‐sensitive species such as green algae. Our laboratory results show that green algae degrade H_2_O_2_ much faster than cyanobacteria. Consequently, the cyanobacterium *Microcystis* was able to survive at higher H_2_O_2_ concentrations in mixtures with the green alga *Chlorella* than in monoculture. Interestingly, even the lysate of destructed *Chlorella* was capable to protect *Microcystis*, indicating a two‐component H_2_O_2_ degradation system in which *Chlorella* provided antioxidant enzymes and *Microcystis* the reductants. The level of interspecific protection provided to *Microcystis* depended on the density of *Chlorella*. These findings have implications for the mitigation of toxic cyanobacterial blooms, which threaten the water quality of many eutrophic lakes and reservoirs worldwide. In several lakes, H_2_O_2_ has been successfully applied to suppress cyanobacterial blooms. Our results demonstrate that high densities of green algae can interfere with these lake treatments, as they may rapidly degrade the added H_2_O_2_ and thereby protect the bloom‐forming cyanobacteria.

## Introduction

Some species play a key role in ecological communities by protecting other species against harmful conditions. For example, the marine heterotrophic bacterium *Alteromonas* sp. protects the cyanobacterium *Prochlorococcus* sp. against cell damage caused by oxidative stress (Morris *et al*., 2008, 2011). This example has led to the Black Queen Hypothesis, an evolutionary theory that refers to the card game ‘Hearts’ and assumes that some species have lost a costly function (the Black Queen) (Morris *et al*., 2012; Mas *et al*., 2016). This function is essential for survival, however, and is still carried out by other species known as helpers. In this case, *Prochlorococcus* lacks a large number of antioxidant genes in comparison to other microorganisms (Bernroitner *et al*., 2009; Morris *et al*., 2012). However, *Alteromonas* acts as a helper by degrading H_2_O_2_ and other reactive oxygen species (ROS) and thereby provides protection to nearby *Prochlorococcus* cells. Similar interspecific protection against oxidative stress might be more widespread than previously recognized (Jakubovics *et al*., 2008; Bobadilla Fazzini *et al*., 2010; Zinser, 2018).

High sensitivity of cyanobacteria to oxidative stress has not only been reported for *Prochlorococcus* in the oligotrophic ocean but also for freshwater cyanobacteria in eutrophic lakes (Drábková *et al*., 2007a; Matthijs *et al*., 2012; Barrington *et al*., 2013; Lürling *et al*., 2014; Weenink *et al*., 2015; Daniel *et al*., 2019). Some of these cyanobacteria can form dense and often toxic blooms during warm periods in summer, deteriorating water quality with severe negative impacts on drinking water reservoirs, irrigation and recreation (Chorus and Bartram, 1999; Carmichael, 2001; Codd *et al*., 2005; O'Neil *et al*., 2012; Huisman *et al*., 2018). Different methods have been developed to suppress cyanobacterial dominance (Ibelings *et al*., 2016; Paerl *et al*., 2018). One promising approach is based on the observation that bloom‐forming cyanobacteria tend to be more sensitive to H_2_O_2_ than many eukaryotic phytoplankton taxa including green algae, diatoms and dinoflagellates (Barroin and Feuillade, 1986; Drábková *et al*., 2007a; Barrington and Ghadouani, 2008; Matthijs *et al*., 2012; Weenink *et al*., 2015; Yang *et al*., 2018; Wang *et al*., 2019; Lusty and Gobler, 2020), most likely because cyanobacteria display a lower antioxidant activity than eukaryotic algae (Matthijs *et al*., 2016). Hence, low concentrations of H_2_O_2_ have been successfully applied to suppress harmful cyanobacterial blooms in lakes (Matthijs *et al*., 2012, 2016) and wastewater ponds (Barrington *et al*., 2013). The applied H_2_O_2_ concentrations (1–5 mg L^−1^; Matthijs *et al*., 2012, 2016) are two to three orders of magnitude higher than natural H_2_O_2_ concentration in lakes (1–50 μg L^−1^; Cooper and Lean, 1989; Häkkinen *et al*., 2004; Cory *et al*., 2017). Yet, the applied H_2_O_2_ concentrations are still far below the H_2_O_2_ sensitivity thresholds of aquatic macroinvertebrates and fish (Rach *et al*., 1997; Gaikowski *et al*., 1999; Matthijs *et al*., 2012; Burson *et al*., 2014), although some zooplankton taxa are sensitive to H_2_O_2_ concentrations in the applied range (Meinertz *et al*., 2008; Matthijs *et al*., 2012; Reichwaldt *et al*., 2012; Yang *et al*., 2018). Hence, this mitigation method acts selectively against cyanobacteria, while environmental impacts on most of the eukaryotic organisms in the ecosystem are kept to a minimum. Furthermore, in comparison to other algicides, application of H_2_O_2_ has the advantage that it breaks down to water and oxygen within a few days, and therefore leaves no long‐term chemical traces in the environment (Matthijs *et al*., 2012).

It is conceivable, however, that the less H_2_O_2_ sensitive eukaryotic phytoplankton act as helpers that degrade the added H_2_O_2_ and thereby protect the targeted cyanobacteria. If so, this would provide a freshwater example of interspecific protection akin to the Black Queen Hypothesis, with practical implications for the mitigation of cyanobacterial blooms. To our knowledge, examples of interspecific protection against oxidative stress have not yet been described in lakes.

To investigate our hypothesis that eukaryotic phytoplankton can protect cyanobacteria against oxidative stress, we performed a series of laboratory experiments with freshwater cyanobacteria and green algae. First, we studied interspecific differences in H_2_O_2_ sensitivity and H_2_O_2_ degradation capacity between three common bloom‐forming cyanobacteria (*Microcystis*, *Anabaena* and *Planktothrix*) and six common green algae (*Chlorella*, *Desmodesmus*, *Kirchneriella*, *Ankistrodesmus*, *Monoraphidium* and *Chlamydomonas*) in monoculture experiments exposed to different H_2_O_2_ concentrations. Subsequently, we applied different H_2_O_2_ concentrations to mixtures of *Microcystis* and *Chlorella* to investigate whether H_2_O_2_ degradation by green algae enhanced the survival of the cyanobacteria. We also examined whether H_2_O_2_ degradation by green algae is mediated by extracellular or intracellular antioxidant activity. Finally, to assess the relevance of these laboratory findings for the mitigation of cyanobacterial blooms in lakes, we compared the effectiveness of H_2_O_2_ treatments in two lakes with different relative abundances of cyanobacteria and green algae.

## Results

### Experiment 1: Cyanobacteria are more sensitive to H_2_O_2_ than green algae

**Fig. 1:**
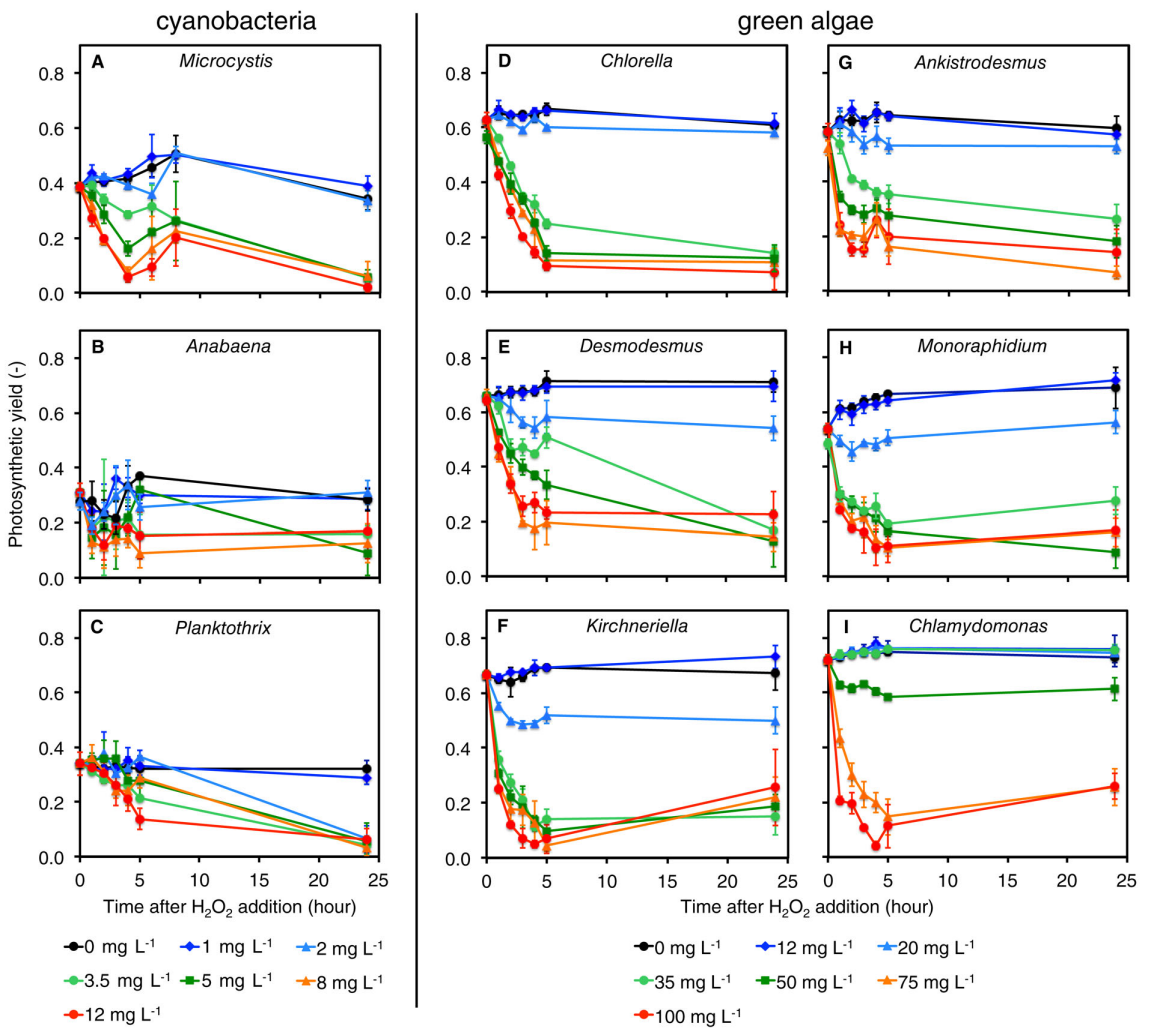
Photosynthetic yield in monocultures of three species of freshwater cyanobacteria and six species of green algae after addition of different H_2_O_2_ concentrations. Cyanobacteria: (A) *Microcystis*, (B) *Anabaena* and (C) *Planktothrix*. Green algae: (D) *Chlorella*, (E) *Desmodesmus*, (F) *Kirchneriella*, (G) *Ankistrodesmus*, (H) *Monoraphidium* and (I) *Chlamydomonas*. Note that the added H_2_O_2_ concentrations were 10‐fold lower in monocultures of the cyanobacteria than in those of the green algae, because the cyanobacteria were more sensitive to H_2_O_2_. Data show mean ± standard deviation (*n* = 3 per data point).

The cyanobacteria *Microcystis*, *Anabaena* and *Planktothrix* differed in their sensitivity to H_2_O_2_ (Fig. 1A–C). The photosynthetic yield of *Microcystis* and *Anabaena* was not affected at H_2_O_2_ concentrations of 1 and 2 mg L^−1^ but strongly declined after the addition of ≥3.5 mg L^−1^ of H_2_O_2_ (Fig. 1A and B). The cyanobacterium *Planktothrix* showed the highest sensitivity. Its photosynthetic yield was not affected at 1 mg L^−1^ of H_2_O_2_ but strongly declined after the addition of ≥2 mg L^−1^ of H_2_O_2_ (Fig. 1C).

The six species of green algae were all much more resistant to H_2_O_2_ than the investigated cyanobacteria. The photosynthetic yield of the green algae was not affected by H_2_O_2_ concentrations up to 12 mg L^−1^ and was mildly suppressed in some taxa after the addition of 20 mg L^−1^ (Fig. 1D–I). The photosynthetic yield of *Chlorella*, *Desmodesmus*, *Kirchneriella*, *Ankistrodesmus* and *Monoraphidium* strongly declined after the addition of ≥35 mg L^−1^ H_2_O_2_ (Fig. 1D–H), whereas the photosynthetic yield of *Chlamydomonas* resisted even higher H_2_O_2_ concentrations and was strongly suppressed only after the addition of ≥75 mg L^−1^ (Fig. 1I). The underlying data of the minimum fluorescence (*F*_0_) and maximum fluorescence (*F*_m_) of the species are reported in Figs S1 and S2.

**Fig. 2:**
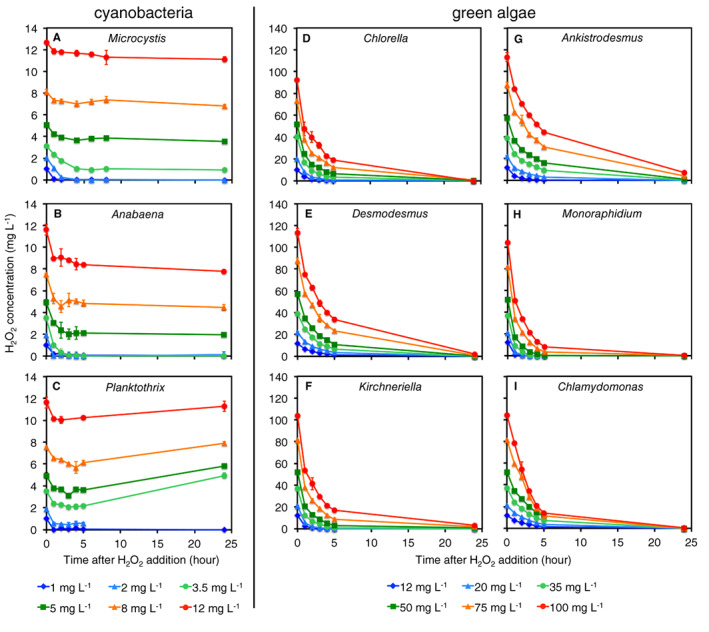
H_2_O_2_ degradation in monocultures of three species of freshwater cyanobacteria and six species of green algae after addition of different H_2_O_2_ concentrations. Cyanobacteria: (A) *Microcystis*, (B) *Anabaena* and (C) *Planktothrix*. Green algae: (D) *Chlorella*, (E) *Desmodesmus*, (F) *Kirchneriella*, (G) *Ankistrodesmus*, (H) *Monoraphidium* and (I) *Chlamydomonas*. The green algal species degrade the added H_2_O_2_ faster than the cyanobacteria. Note the different scales of the *y*‐axes: the added H_2_O_2_ concentrations were 10‐fold lower in monocultures of the cyanobacteria than in those of the green algae. Data show mean ± standard deviation (*n* = 3 per data point).

The H_2_O_2_ degradation capacity was much higher in axenic monocultures of the green algae than in those of the cyanobacteria (Fig. 2). All three cyanobacteria completely degraded 1 mg L^−1^ of H_2_O_2_ within a few hours, *Microcystis* and *Anabaena* also completely degraded 2 mg L^−1^ of H_2_O_2_, and *Anabaena* completely degraded 3.5 mg L^−1^ (Fig. 2A–C). However, H_2_O_2_ added at higher concentrations was only partially degraded and still remained in the cyanobacterial cultures after 24 h. In contrast, all six species of green algae completely degraded even the highest H_2_O_2_ concentration of 100 mg L^−1^ within 24 h (Fig. 2D–I).

### Experiment 2: *Chlorella* protects *Microcystis* against H_2_O_2_

**Fig. 3:**
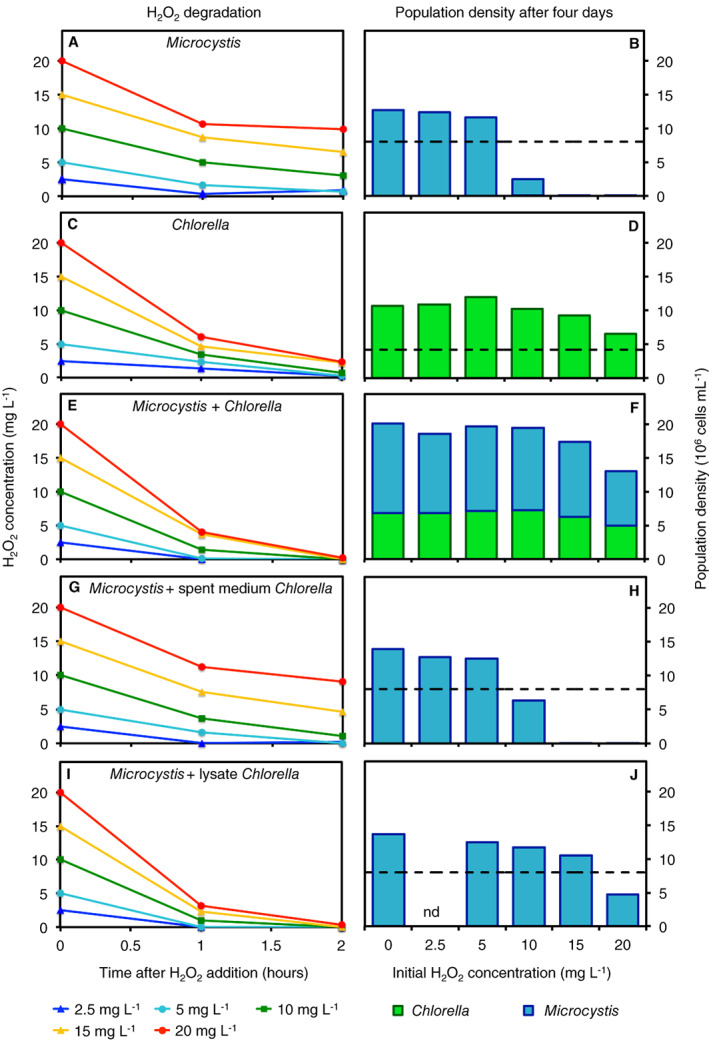
The green alga *Chlorella* protects the cyanobacterium *Microcystis* against high H_2_O_2_ concentrations. H_2_O_2_ was added at different initial concentrations to (A, B) *Microcystis* monocultures, (C, D) *Chlorella* monocultures, (E, F) mixtures of *Microcystis* and *Chlorella*, (G, H) mixtures of *Microcystis* with spent medium of *Chlorella* and (I, J) mixtures of *Microcystis* with the lysate of destructed *Chlorella* cells. Left panels show H_2_O_2_ degradation during the first 2 h after the addition of different H_2_O_2_ concentrations (2.5, 5, 10, 15 and 20 mg L^−1^). Right panels show cell counts of *Microcystis* (blue bars) and *Chlorella* (green bars) after 4 days. nd = not determined. Horizontal dashed lines indicate the initial population densities of *Microcystis* (panels B, H and J) and *Chlorella* (panel D) when the H_2_O_2_ was added (at *t* = 0). Initial population densities of *Microcystis* and *Chlorella* in panel F were the same as in panels B and D respectively.

**Fig. 4:**
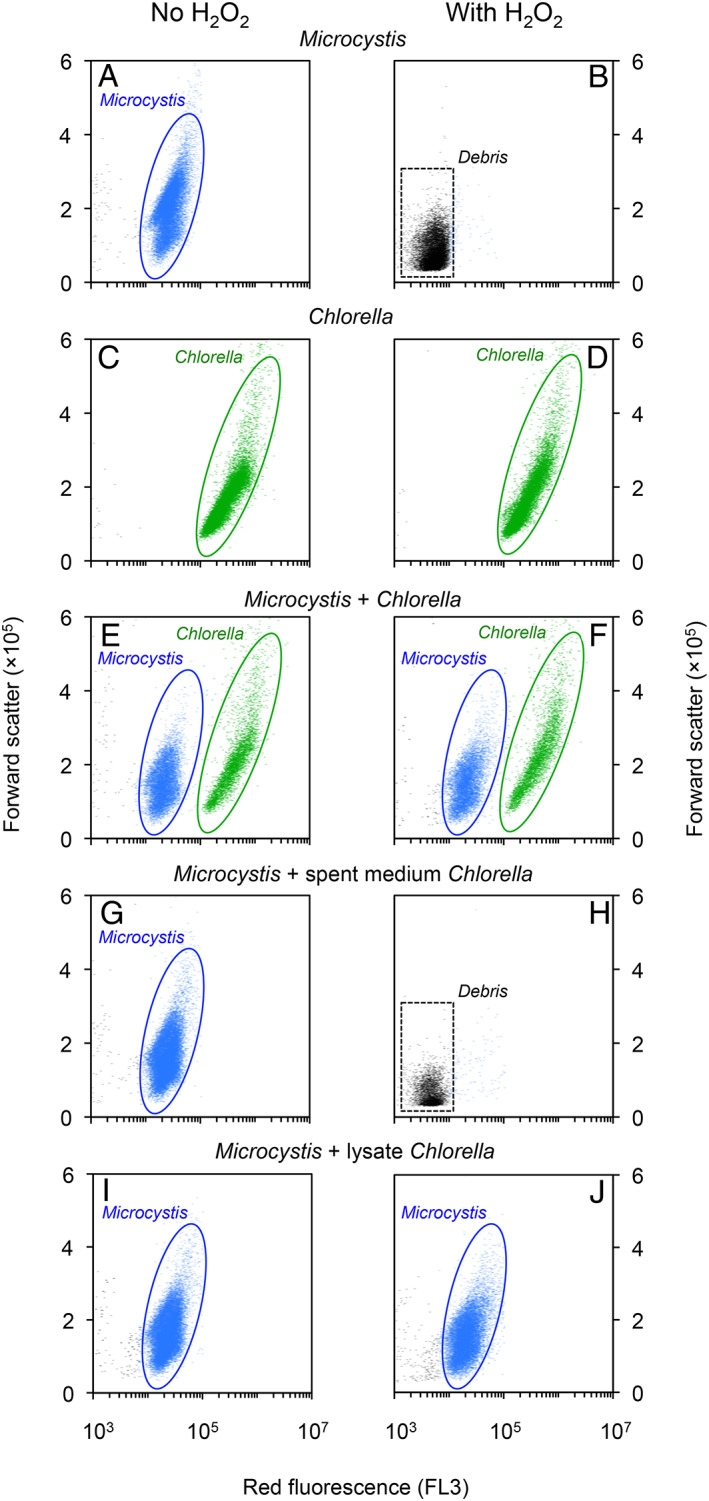
Flow cytometer analyses illustrating protection of the cyanobacterium *Microcystis* by the green alga *Chlorella*. Left panels show flow cytometer counts of the control treatments (0 mg L^−1^ of H_2_O_2_) and right panels show the results 4 days after addition of 15 mg L^−1^ of H_2_O_2_. (A, B) *Microcystis* monocultures, (C, D) *Chlorella* monocultures, (E, F) mixtures of *Microcystis* and *Chlorella*, (G, H) mixtures of *Microcystis* with spent medium of *Chlorella*, and (I, J) mixtures of *Microcystis* with the lysate of destructed *Chlorella* cells. Each dot in the scatterplots represents a particle passing the detection unit of the flow cytometer. Forward scatter (FSC‐A) and red fluorescence emission (670 nm, FL3) are used to discriminate between *Microcystis* and *Chlorella* cells (encircled areas). Dots with low forward scatter and red fluorescence in the lower‐left corner of (B) and (H) represent cellular debris.

As a next step, we performed monoculture and mixture experiments with *Microcystis* and *Chlorella* at six H_2_O_2_ concentrations to investigate the potential for interspecific protection (Fig. 3). Monoculture experiments confirmed that *Chlorella* degraded H_2_O_2_ faster than *Microcystis* (Fig. 3A and C), in agreement with the previous results (Fig. 2). *Microcystis* monocultures were strongly suppressed after H_2_O_2_ addition of 10 mg L^−1^ and collapsed at 15 and 20 mg L^−1^ (Fig. 3B). Flow cytometer counts illustrate that *Microcystis* cells exposed to these high H_2_O_2_ concentrations were destroyed to cellular debris (Fig. 4A and B). Conversely, *Chlorella* monocultures sustained growth at all applied H_2_O_2_ additions (Figs 3D and Fig. 4C,D).

In mixtures of *Microcystis* and *Chlorella*, H_2_O_2_ degradation was even slightly faster than in the monoculture experiments of *Chlorella* and both species sustained growth even at the high H_2_O_2_ additions of 15 and 20 mg L^−1^ (Figs 3E,F and 4E,F). Note that, at all applied H_2_O_2_ concentrations, *Chlorella* reached a consistently lower population density in mixtures with *Microcystis* (Fig. 3F) than in the *Chlorella* monoculture (Fig. 3D), indicating that *Microcystis* was an effective competitor restraining the population density of *Chlorella*.

When *Microcystis* was grown in a mineral medium to which we added spent medium of the *Chlorella* cultures, the H_2_O_2_ degradation rate was comparable to that in the *Microcystis* monoculture (compare Fig. 3G and A) and the *Microcystis* population collapsed at H_2_O_2_ additions of 15 and 20 mg L^−1^ (Figs 3H and 4G,H). However, when *Microcystis* was grown in mineral medium to which we added lysate of the *Chlorella* cultures, containing the cellular debris of *Chlorella*, the H_2_O_2_ degradation rate was comparable to that in the mixture of *Microcystis* and *Chlorella* cells (compare Fig. 3I and E) and the *Microcystis* population sustained growth even at the high H_2_O_2_ additions of 15 and 20 mg L^−1^ (Figs 3J and 4I,J).

### Experiment 3: Protection by *Chlorella* is density‐dependent

**Fig. 5:**
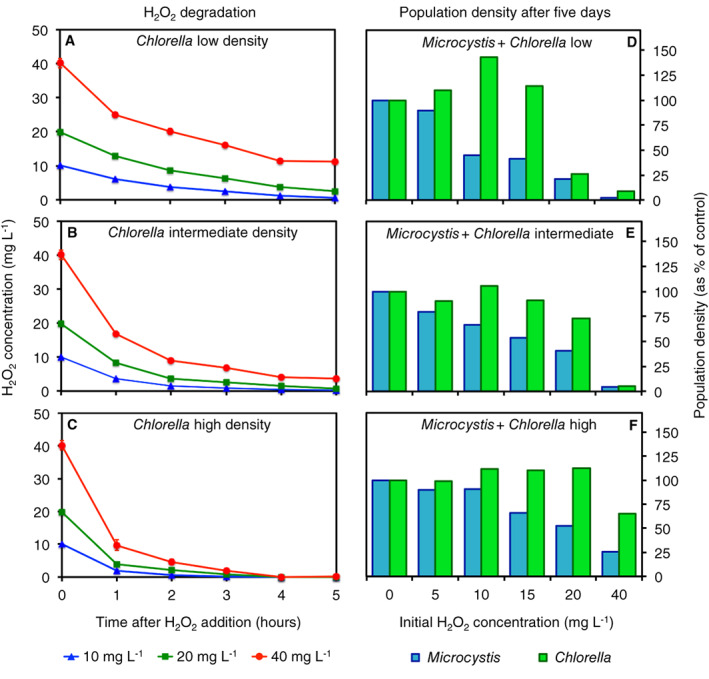
Protection of the cyanobacterium *Microcystis* against H_2_O_2_ depends on the population density of the green alga *Chlorella*. (A–C) Degradation of added H_2_O_2_ (10, 20 and 40 mg L^−1^) in monocultures with (A) low, (B) intermediate and (C) high initial population densities of *Chlorella*. Data show mean ± standard deviation (*n* = 3 per data point). (D–F) Population densities (expressed as % of the control) of *Microcystis* and *Chlorella* at 5 days after the addition of different H_2_O_2_ concentrations to mixtures of *Microcystis* with (D) low, (E) intermediate and (F) high initial population densities of *Chlorella*. The initial *Microcystis* density was the same in (D)–(F).

H_2_O_2_ degradation was faster in high‐density *Chlorella* cultures than in low‐density *Chlorella* cultures (Fig. 5A–C). To investigate whether this density‐dependent H_2_O_2_ degradation affects interspecific protection, we performed experiments with *Microcystis* and three different population densities of *Chlorella* (Fig. 5D–F). In mixed cultures with low *Chlorella* densities, *Microcystis* sustained population densities at 45% and 21% of the control after addition of 10 and 20 mg L^−1^ of H_2_O_2_ respectively but was suppressed to <3% of the control at 40 mg L^−1^ of H_2_O_2_ (Fig. 5D). At intermediate *Chlorella* densities, *Microcystis* sustained higher population densities (67% and 40% of the control respectively) at 10 and 20 mg L^−1^ of H_2_O_2_ but was still suppressed to <5% of the control at 40 mg L^−1^ of H_2_O_2_ (Fig. 5E). At high *Chlorella* densities, *Microcystis* sustained population densities that were 90%, 53% and 25% of the control at 10, 20 and 40 mg L^−1^ of H_2_O_2_ respectively (Fig. 5F). Hence, *Microcystis* survival improved with increasing population densities of *Chlorella*.

### Experiment 4: Rapid H_2_O_2_ degradation is mediated by living cells

In the absence of living cells, the H_2_O_2_ degradation rate by the lysate and spent medium of both species was very low (Fig. S1). In particular, the lysate of *Chlorella* by itself hardly degraded any H_2_O_2_ (Fig. S1a), whereas the lysate of *Chlorella* did show a fast H_2_O_2_ degradation rate in the presence of *Microcystis* cells (Fig. 3I).

### Lake treatments

**Fig. 6:**
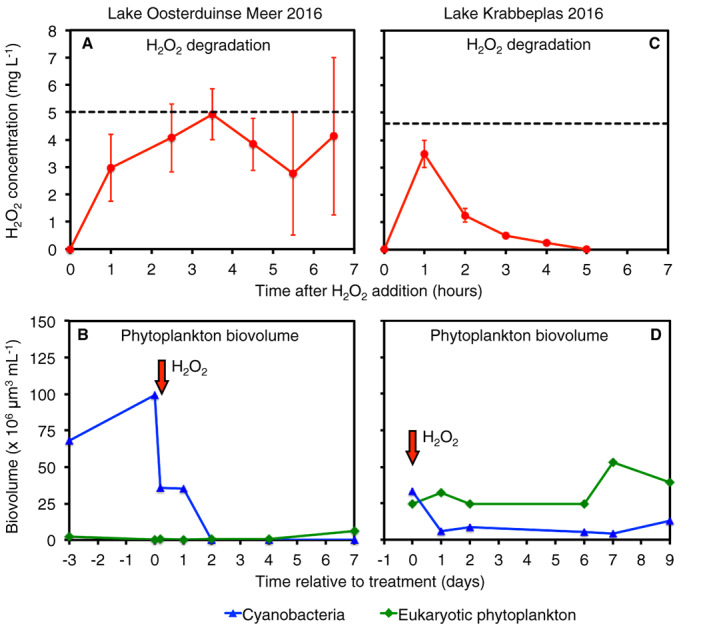
H_2_O_2_ treatments of two lakes with different relative abundances of cyanobacteria and eukaryotic phytoplankton. (A, B) Lake Oosterduinse Meer was dominated by cyanobacteria (99.6%) with only a minor contribution by eukaryotic phytoplankton (0.35%) prior to the treatment. In this lake, (A) the added H_2_O_2_ remained in the lake for several hours, and (B) the cyanobacterial bloom was effectively suppressed. (C, D) Lake Krabbeplas consisted of a mixture of cyanobacteria (57.5%) and eukaryotic phytoplankton (42.5%). In this lake, (C) the added H_2_O_2_ concentration declined much faster, and (D) the cyanobacteria were much less suppressed than in Lake Oosterduinse Meer. Data in (A) and (C) show mean ± standard error (*n* = 2–10 per data point); the horizontal dashed line indicates the target H_2_O_2_ concentration (i.e. the expected concentration based on the total amount of H_2_O_2_ added to the lake). Red arrows in (B) and (D) indicate the start of H_2_O_2_ addition. See Tables S1 and S2 for detailed changes in phytoplankton community composition.

Lake Oosterduinse Meer was dominated by a dense *Aphanizomenon* bloom in June 2016 (Fig. 6A and B). On the day of the H_2_O_2_ treatment, prior to H_2_O_2_ addition, the lake contained a cyanobacterial biovolume of 99.0 × 10^6^ μm^3^ ml^−1^ (99.6% of total phytoplankton), consisting almost entirely of *Aphanizomenon flos‐aquae* (99.6%) with minor contributions by *Microcystis* spp. (0.02%) and *Dolichospermum* spp. (0.01%). Eukaryotic phytoplankton contributed only 0.35 × 10^6^ μm^3^ ml^−1^ (0.35%), consisting mainly of green algae (0.17%) and diatoms (0.11%) (Table S1).

During the treatment of Lake Oosterduinse Meer, the target H_2_O_2_ concentration of 5.0 mg L^−1^ was reached ~3 h after the H_2_O_2_ addition started (Fig. 6A). High H_2_O_2_ concentrations ≥2.5 mg L^−1^ were maintained for several hours, although the added H_2_O_2_ had disappeared after 24 h. The cyanobacterial biovolume decreased by 64.0% (relative to the initial cyanobacterial biovolume) at 5 h after the start of the H_2_O_2_ addition and almost completely disappeared 2 days later (>99.8% decline) (Fig. 6B). The cyanobacterial biovolume remained very low (<0.02%) during the first week after the treatment, while eukaryotic phytoplankton (including the green algae) increased almost 20‐fold.

Lake Krabbeplas consisted of a mixture of cyanobacteria and eukaryotic phytoplankton (Fig. 6C and D). On the treatment day prior to H_2_O_2_ addition, Lake Krabbeplas contained a cyanobacterial biovolume of 33.2 × 10^6^ μm^3^ ml^−1^ (57.5% of total phytoplankton), including *Limnococcus limneticus* (29.1%), *Pseudanabaena limnetica* (28.1%), *Planktothrix agardhii* (0.8%) and *Coelomoron pusillum* (0.6%). Eukaryotic phytoplankton was slightly less abundant than the cyanobacteria, with a eukaryotic biovolume of 24.5 × 10^6^ μm^3^ ml^−1^ (42.5%) consisting of a rich diversity of green algae (17.1%) including *Monoraphidium contortum*, *Desmodesmus* spp. and *Chlamydomonas* spp., and other taxa such as diatoms, dinoflagellates and cryptomonads (Table S2).

In Lake Krabbeplas, H_2_O_2_ degradation was much faster than in Lake Oosterduinse Meer (Fig. 6C). Although the total amount of H_2_O_2_ administered to the lake was equivalent to 4.6 mg L^−1^, the maximum H_2_O_2_ concentration measured in the lake was only 3.5 mg L^−1^. Within 2 h after the start of the treatment, the H_2_O_2_ concentration in Lake Krabbeplas had decreased to <2 mg L^−1^, and within 5 h all added H_2_O_2_ had disappeared. One day after the treatment, the cyanobacterial biovolume had declined by 82.1% (relative to the initial cyanobacterial biovolume). Contrary to Lake Oosterduinse Meer, however, cyanobacteria in Lake Krabbeplas did not decline further but remained present at a stable biovolume of ~6 × 10^6^ μm^3^ ml^−1^ during the first week after the treatment and subsequently increased (Fig. 6D).

## Discussion

### Differences in H_2_O_2_ sensitivity between cyanobacteria and green algae

Oxygenic phototrophic organisms have developed antioxidant defences to degrade ROS and, thereby, to protect themselves against oxidative damage (Bernroitner *et al*., 2009; Latifi *et al*., 2009; Dietz, 2011; Foyer and Shigeoka, 2011). Our results show considerable interspecific variation in H_2_O_2_ sensitivity among cyanobacteria and green algae representative of eutrophic lakes (Figs 1 and 2). Among the cyanobacteria, *Planktothrix* was more sensitive than *Microcystis* and *Anabaena*. This result aligns with other recent studies in which *Planktothrix* was more sensitive to H_2_O_2_ than *Microcystis* (Yang *et al*., 2018; Lusty and Gobler, 2020). Among the green algae, *Chlamydomonas reinhardtii* was more resistant than the five other taxa that we investigated. Similarly, Drábková *et al*. (2007a) reported that a *C*. *reinhardtii* strain was more H_2_O_2_ resistant than the green algae *Scenedesmus quadricauda* and *Pseudokirchneriella subcapitata*.

However, the most striking difference in sensitivity was observed between cyanobacteria and green algae. Our results show that, in monoculture, the freshwater cyanobacteria were all more sensitive to H_2_O_2_ than the green algae. The photosynthetic yield of the bloom‐forming cyanobacterium *Planktothrix* collapsed at ≥2 mg L^−1^ H_2_O_2_ and *Microcystis* and *Anabaena* at ≥3.5 mg L^−1^ H_2_O_2_, whereas the investigated green algae were hardly affected up to at least 20 mg L^−1^ H_2_O_2_ (Fig. 1). These results are in agreement with previous studies, which have also shown that cyanobacteria are more sensitive to H_2_O_2_ than eukaryotic phytoplankton including green algae (Barroin and Feuillade, 1986; Drábková *et al*., 2007a; Drábková *et al*., 2007b; Matthijs *et al*., 2012; Weenink *et al*., 2015; Yang *et al*., 2018; Wang *et al*., 2019; Lusty and Gobler, 2020).

The difference in H_2_O_2_ sensitivity between cyanobacteria and green algae appears to be related to differences in H_2_O_2_ degradation rates, since all six species of green algae degraded the added H_2_O_2_ much faster than the cyanobacterial species (Fig. 2). This might be explained by a key difference in photophysiology between cyanobacteria and eukaryotic algae (Helman *et al*., 2003; Allahverdiyeva *et al*., 2015; Matthijs *et al*., 2016). When eukaryotic phototrophs are exposed to high light or low inorganic carbon concentrations, excess electrons generated by photosynthesis are transferred to oxygen (O_2_) producing the superoxide anion (O_2_^−^) via the Mehler reaction (Mehler, 1951; Asada, 1999). The highly reactive superoxide is rapidly converted to H_2_O_2_ by superoxide dismutase, which is subsequently converted to water by peroxidases and catalases to protect the cells against oxidative damage (Asada, 1999; Shigeoka *et al*., 2002; Apel and Hirt, 2004). In contrast, cyanobacteria deploy a ‘Mehler‐like reaction’, in which excess electrons are transferred to oxygen by the flavodiiron proteins Flv1 and Flv3, which produce water directly without intermediary production of O_2_^−^ and H_2_O_2_ (Helman *et al*., 2003; Allahverdiyeva *et al*., 2015). This implies that cyanobacteria will produce much less H_2_O_2_ during photosynthesis than eukaryotic phytoplankton. Accordingly, in evolutionary terms, cyanobacteria may not have had the need for a similarly high H_2_O_2_ degradation capacity as eukaryotic phytoplankton.

### Green algae protect cyanobacteria against high oxidative stress

Our results show that, in combination with the green alga *Chlorella*, the cyanobacterium *Microcystis* can survive at much higher H_2_O_2_ concentrations than in monoculture, at least up to 20 mg L^−1^ of H_2_O_2_ (Figs 3 and 4). Apparently, the high H_2_O_2_ degradation rate by *Chlorella* protects *Microcystis* against oxidative stress.

In several ways, these results resemble the previously described example of protection against oxidative stress of *Prochlorococcus* by *Alteromonas* in the oligotrophic ocean (Morris *et al*., 2011, 2012). For example, our results show that degradation of H_2_O_2_ is essential for survival, and that *Microcystis* relies on the H_2_O_2_ degradation activity of *Chlorella* when H_2_O_2_ concentrations become high. Using the terminology of the Black Queen Hypothesis, the green alga serves as helper and the cyanobacterium as the beneficiary.

There are also some interesting differences, however. In particular, the observed interspecific protection of freshwater bloom‐forming cyanobacteria by green algae occurs at much higher H_2_O_2_ concentrations than the protection of the marine cyanobacterium *Prochlorococcus* by *Alteromonas*. Marine *Prochlorococcus* spp. lack most antioxidant enzymes, and therefore they are very sensitive to H_2_O_2_ (Morris *et al*., 2012). Experiments have shown that *Alteromonas* may already provide protection to *Prochlorococcus* at H_2_O_2_ concentrations as low as 0.007 mg L^−1^ (Morris *et al*., 2011), which is of similar magnitude as the natural H_2_O_2_ concentration in surface waters of the oligotrophic open ocean (Yuan and Shiller, 2001; Gerringa *et al*., 2004; Yuan and Shiller, 2005). In contrast, most freshwater cyanobacteria, including *Microcystis*, contain genes encoding anti‐ROS enzymes such as peroxiredoxins (Franguel *et al*., 2008; Bernroitner *et al*., 2009; Schuurmans *et al*., 2018). Our results show that these bloom‐forming cyanobacteria are much less H_2_O_2_ sensitive than *Prochlorococcus*. Without interspecific protection, they can survive up to 1–2 mg L^−1^ of H_2_O_2_ (Fig. 1; see also Drábková *et al*., 2007a; Barrington *et al*., 2013; Lürling *et al*., 2014; Piel *et al*., 2020). For this reason, the H_2_O_2_ concentrations applied in lake treatments greatly exceed the H_2_O_2_ concentrations that they would naturally encounter in lakes (0.001–0.050 mg L^−1^; Cooper and Lean, 1989; Häkkinen *et al*., 2004; Cory *et al*., 2017). Although the protective mechanism shows many similarities with the *Prochlorococcus*‐*Alteromonas* example of the Black Queen Hypothesis, it is thus unlikely that protection of bloom‐forming cyanobacteria by green algae has developed from a co‐evolutionary interaction between the species.

Furthermore, the ecological relationship between helper and beneficiary is different. Although reciprocity is not required for the Black Queen Hypothesis, the interaction between *Alteromonas* and *Prochlorococcus* might be beneficial for both species. The heterotrophic bacterium *Alteromonas* provides protection against extracellular H_2_O_2_ to *Prochlorococcus* cells, while the phototrophic *Prochlorococcus* in turn fixes atmospheric carbon that partly becomes available as a public good (Morris *et al*., 2012). A major novelty of our study is that one phototrophic species protects another phototrophic species against oxidative stress. In our experiments, *Chlorella* protects *Microcystis* but *Microcystis* does not seem to return a favour to *Chlorella*. Instead, cyanobacteria and green algae compete with each other for limiting resources, and our data suggest that the survival of *Microcystis* may even suppress the *Chlorella* population to some extent (as indicated by the difference in population densities of *Chlorella* between Fig. 3D and F). In a sense, *Chlorella* facilitates its competitor.

### Which card is being played?

The antioxidant activity of *Chlorella* is essential for H_2_O_2_ degradation and hence for the interspecific protection of *Microcystis*. Interestingly, the lysate of *Chlorella* by itself caused a very slow H_2_O_2_ degradation (Fig. S1), whereas the lysate of *Chlorella* in combination with *Microcystis* cells enabled a much faster H_2_O_2_ degradation (Fig. 3I). Apparently, *Microcystis* can activate the H_2_O_2_ degradation activity in lysate of *Chlorella*. Moreover, in the presence of *Microcystis* cells, the lysate of *Chlorella* had a much higher H_2_O_2_ degradation rate than spent medium of *Chlorella* (Fig. 3G and I). The latter result indicates that the H_2_O_2_‐degrading enzymes are contained in the *Chlorella* cells rather than being exported extracellularly. So, what is the expensive Black Queen card that *Chlorella* holds by which *Microcystis* is protected, and what essential component for H_2_O_2_ degradation may be missing in the lysate of destructed *Chlorella* cells that is provided by *Microcystis*?

Our results suggest that H_2_O_2_ degradation in this system requires two components. The cells of *Chlorella* likely contain H_2_O_2_‐degrading enzymes. These enzymes function well in living *Chlorella* cells (Fig. 3C and D). The enzymes can also function in the lysate (Figs 3I,J and 4I,J), but there they cannot degrade H_2_O_2_ by themselves (Fig. S1). Apparently, a second compound is required that fuels H_2_O_2_ degradation, most likely a flow of reductants needed by the enzymes for H_2_O_2_ reduction (e.g. Shigeoka *et al*., 2002). These reductants can be provided by living *Chlorella* cells, but our results indicate that in the absence of living *Chlorella* cells the provision of reductants can also be taken over by *Microcystis* (Figs 3I,J and 4I,J).

*Microcystis* PCC 7806 does not produce catalase (Franguel *et al*., 2008; Schuurmans *et al*., 2018). Whether *Chlorella sorokiniana* 211‐8k produces catalase is not known. However, catalase can scavenge large amounts of H_2_O_2_ without the requirement for an electron‐donating substrate (Apel and Hirt, 2004), which does not fit the two‐component system observed in our data. Moreover, in our experiments the cell‐free lysate of *Chlorella* had a very low H_2_O_2_ degradation rate (Fig. S1), suggesting the absence or only a low potency of catalase mediated anti‐ROS activity in *Chlorella sorokiniana* 211‐8k.

**Fig. 7:**
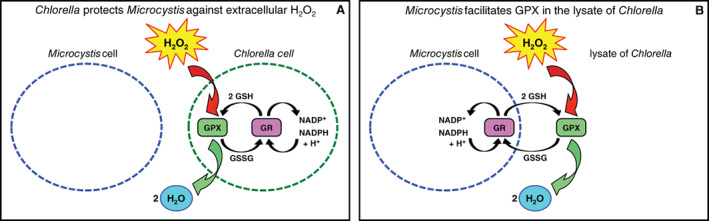
The proposed mechanism by which the green alga *Chlorella* protects the cyanobacterium *Microcystis* against oxidative stress. A. In living *Chlorella* cells, extracellular H_2_O_2_ diffuses into the cells where peroxidases (e.g. GPX) utilize reductants (reduced glutathione, GSH) as electron donor to convert H_2_O_2_ into water. Oxidized glutathione (GSSG) is regenerated to GSH by the glutathione reductase (GR) cycle, which requires NADPH. B. In the lysate of *Chlorella*, peroxidases (e.g. GPX) can still operate thanks to reductants (e.g. GSH) produced and exported by living *Microcystis* cells. In this case, *Microcystis* actively facilitates its own protection by enabling the degradation of H_2_O_2_ by the peroxidases produced earlier by *Chlorella*.

A more plausible explanation is that *Chlorella* contains glutathione peroxidase (GPX) and/or ascorbate peroxidase (APX) to scavenge H_2_O_2_. In case of GPX, H_2_O_2_ is reduced to H_2_O by oxidizing glutathione (GSH) to glutathione disulfide (GSSG). Subsequently, GSSG is regenerated to GSH by the glutathione‐reductase cycle, which requires an influx of reductants from, e.g. NADPH. APX works in a similar way using ascorbate as reductant, which is coupled to glutathione via the glutathione‐ascorbate cycle. Many *Chlorella* strains including *Chlorella sorokiniana* 211‐8k contain GPX and/or APX, usually in combination with glutathione reductase (Pelah and Cohen, 2005; Wang and Xu, 2012; Salbitani *et al*., 2015). By contrast, sequencing of the genome of *Microcystis* PCC 7806 did not reveal GPX or APX, but it does contain glutathione synthetase and reductase to produce and regenerate glutathione (Franguel *et al*., 2008; Straub *et al*., 2011; Zilliges *et al*., 2011). Glutathione can be actively exported to and imported from the extracellular environment in both its reduced (GSH) and oxidized (GSSG) forms (Couto *et al*., 2016). Hence, a plausible hypothesis for our results is that the high H_2_O_2_‐degrading activity of peroxidases produced by *Chlorella* protects both *Chlorella* and *Microcystis* against oxidative stress (Fig. 7A). When *Chlorella* cells were destructed, their peroxidase enzymes remained intact in the lysate but now their activity required the delivery of reductants by *Microcystis* (Fig. 7B). Further research may help to elucidate this mechanism in full detail, for instance by investigating whether peroxidases such as GPX or APX are indeed active in *Chlorella*, and whether addition of these peroxidases in pure form to monocultures of *Microcystis* would provide similar protection against oxidative stress as the addition of lysate of *Chlorella*.

### Implications for lake treatments

In several lakes and ponds, H_2_O_2_ addition has been applied as an emergency method to suppress toxic cyanobacterial blooms (Matthijs *et al*., 2012, 2016; Barrington *et al*., 2013). What constitutes a successful treatment? The suppression of toxic cyanobacteria should be strong enough to avoid health risks associated with recreation or drinking water supply. Furthermore, the cyanobacterial bloom should be sufficiently suppressed to prevent rapid regrowth from the remaining cyanobacterial population. In practice, this implies that successful treatments should aim at an almost complete elimination of the cyanobacterial bloom.

As shown by our laboratory results, green algae and presumably also other eukaryotic phytoplankton can counteract the treatment success by protecting cyanobacteria against oxidative stress. Although whole‐lake treatments are expensive and cannot be controlled to a similar degree as laboratory experiments, the results of the H_2_O_2_ treatments in Lake Oosterduinse Meer and Lake Krabbeplas are consistent with these laboratory findings (Fig. 6). In Lake Oosterduinse Meer, the abundance of eukaryotic phytoplankton including green algae was very low, the added H_2_O_2_ concentration remained above 2.5 mg L^−1^ for at least 5.5 h, and the dense cyanobacterial population was completely suppressed by the H_2_O_2_ treatment (Fig. 6A and B). Lake Krabbeplas initially contained less cyanobacteria than Lake Oosterduinse Meer but a much higher abundance of eukaryotic phytoplankton. In this case, the added H_2_O_2_ was degraded rapidly, and a substantial fraction (~15%) of the cyanobacterial population survived the H_2_O_2_ treatment (Fig. 6C and D). Consequently, the H_2_O_2_ treatment was more successful in Lake Oosterduinse Meer than in Lake Krabbeplas.

Similar results were obtained by a recent study by Lusty and Gobler (2020). They applied H_2_O_2_ to incubation experiments with natural phytoplankton assemblages sampled from lakes. Mill Pond was dominated by a high cyanobacterial biomass (expressed as Chl*a* L^−1^), whereas the biomass of green algae was below detectable levels at the start of the experiment. Addition of H_2_O_2_ to incubation experiments containing the phytoplankton community of this lake caused a major collapse of 96% of the cyanobacterial population (their Fig. 4A). In contrast, in Roth Pond the initial green algal biomass was more than twice the cyanobacterial biomass. In this case, H_2_O_2_ addition to the incubation experiments of Roth Pond reduced the cyanobacteria by only 34% (their Fig. 6A). Hence, the results for Mill Pond and Roth Pond obtained by Lusty and Gobler (2020) support our observations that a high abundance of green algae in the phytoplankton community may reduce the efficacy of H_2_O_2_ to suppress cyanobacterial blooms.

The density‐dependent nature of this interspecific protection implies that, at a higher concentration of green algae, a higher H_2_O_2_ dosage will be required to suppress cyanobacterial blooms. One could argue to raise the H_2_O_2_ dosage until the desired collapse of the cyanobacterial population is achieved. From a water management perspective, however, there are limits on the amount of H_2_O_2_ that can be applied to lakes, because high H_2_O_2_ concentrations can be lethal to sensitive non‐target organisms such as zooplankton (Matthijs *et al*., 2012; Reichwaldt *et al*., 2012; Burson *et al*., 2014; Yang *et al*., 2018). For this reason, Matthijs *et al*. (2016) generally recommended H_2_O_2_ concentrations ≤5 mg L^−1^ as a precautionary limit for lake applications. As a consequence, extensive H_2_O_2_ scavenging by high abundances of green algae and other eukaryotic phytoplankton can protect bloom‐forming cyanobacteria against oxidative stress, and thereby prevent successful suppression of toxic cyanobacterial blooms by H_2_O_2_ treatments.

## Experimental procedures

### Species and pre‐culture conditions

Axenic cultures of *Microcystis aeruginosa* strain PCC 7806 (hereafter *Microcystis*), *Planktothrix agardhii* PCC 7811 (*Planktothrix*) and *Anabaena* PCC 7938 (*Anabaena*) were kindly provided by the Institute Pasteur Collection (Paris, France). *Chlorella sorokiniana* SAG 211‐8k, *Kirchneriella contorta* SAG 11.81, *Monoraphidium griffithii* SAG 202‐13, *Chlamydomonas reinhardtii* SAG 77.81, *Desmodesmus armatus* SAG 276‐4e and *Ankistrodesmus falcatus* SAG 202‐9 were all kindly provided by the SAG Culture Collection of Algae (Göttingen, Germany).

Cells were pre‐cultured under axenic conditions in 500 ml Erlenmeyer flasks with 100 ml culture volume on a rotary shaker at 100 rpm, in nutrient‐rich BG‐11 medium (Rippka *et al*., 1979) at 20°C under continuous illumination of 30 μmol photons m^−2^ s^−1^ provided by white fluorescent tubes (TL 34 lamps, Philips, Eindhoven). Inocula for the experiments were taken from the exponential growth phase. Axenic conditions were confirmed by microscopic examination and flow cytometry analysis of the cultures.

### Experiment 1: Sensitivity of cyanobacteria and green algae to H_2_O_2_

In Experiment 1, we tested whether cyanobacteria and green algae would differ in their sensitivity to H_2_O_2_ and H_2_O_2_ degradation capacity. First, we exposed monocultures of the cyanobacteria *Microcystis*, *Anabaena* and *Planktothrix* to different H_2_O_2_ concentrations. For each species, 12‐well plates (Corning, Kennebunk, USA) were inoculated with 4.3 ml aliquots from pre‐cultures with similar biovolumes, to which 0.2 ml of H_2_O_2_ was added at different concentrations. Population biovolumes at the start of the experiment were 0.60 ± 0.05 mm^3^ mL^−1^ (mean ± standard error, *n* = 12). H_2_O_2_ was added to the well plates with cyanobacteria to obtain final H_2_O_2_ concentrations of 0 (control), 1, 2, 3.5, 5, 8 and 12 mg L^−1^.

Subsequently, well plates with monocultures of the green algae *Chlorella*, *Kirchneriella*, *Monoraphidium*, *Chlamydomonas*, *Desmodesmus* and *Ankistrodesmus* with initial population biovolumes of 0.58 ± 0.03 mm^3^ mL^−1^ (mean ± standard error, *n* = 23) were exposed to different H_2_O_2_ concentrations. Because previous studies indicated that green algae are less sensitive to H_2_O_2_ than cyanobacteria (Barroin and Feuillade, 1986; Drábková *et al*., 2007a; Matthijs *et al*., 2012; Weenink *et al*., 2015), we applied 10‐fold higher final H_2_O_2_ concentrations of 0, 12, 20, 35, 50, 75 and 100 mg L^−1^ to the wells with green algae. All treatments were performed with six replicates.

Well plates were put on top of a laboratory bench at room temperature (~20°C), away from direct sunlight and evenly illuminated by fluorescent tubes (TL5‐49 W, Philips) at ~10 μmol photons m^−2^ s^−1^. The wells were carefully mixed by rotary movement with a clean spatula once every hour, except during night‐time. Samples (300 μl) from three replicates were taken at *t* = 0, 1, 2, 3, 4, 5 and 24 h after H_2_O_2_ addition for analysis of the H_2_O_2_ concentration. Simultaneously, photosynthetic yield in the other three replicates was measured by pulse‐amplitude modulation (PAM) fluorometry.

### Experiment 2: Interspecific protection against H_2_O_2_ addition

In Experiment 2, we investigated the response of mixtures of *Microcystis* and *Chlorella* to different H_2_O_2_ concentrations. The experiment was performed in 50 ml incubation flasks and consisted of five different treatments:

(1) *Microcystis* monocultures (5 ml preculture of *Microcystis* and 20 ml BG‐11 medium),

(2) *Chlorella* monocultures (5 ml preculture of *Chlorella* and 20 ml BG‐11 medium),

(3) mixtures of *Microcystis* and *Chlorella* (5 ml preculture of *Microcystis*, 5 ml preculture of *Chlorella* and 15 ml BG‐11 medium),

(4) mixtures of *Microcystis* with spent medium of *Chlorella* (5 ml preculture of *Microcystis*, 5 ml spent medium of *Chlorella* and 15 ml BG‐11 medium),

(5) mixtures of *Microcystis* with the lysate of *Chlorella* cells (5 ml preculture of *Microcystis*, 5 ml lysate of *Chlorella* and 15 ml BG‐11 medium).

At the start of the experiment, 1 ml of diluted H_2_O_2_ was added to the incubation flasks to obtain H_2_O_2_ concentrations of 0 (control), 2.5, 5, 10, 15 and 20 mg L^−1^. Population densities in the incubation flasks at the start of the experiment were 8.0 × 10^6^ cells mL^−1^ for *Microcystis* and 4.2 × 10^6^ cells mL^−1^ for *Chlorella*.

Incubation flasks were put on top of a laboratory bench at room temperature and mixed twice daily, as described above. Samples were taken prior to H_2_O_2_ addition (*t* = 0 h) and at *t* = 1 and 2 h after H_2_O_2_ addition for analysis of the H_2_O_2_ concentration. Population densities were counted 4 days after H_2_O_2_ addition.

For the preparation of spent medium of *Chlorella*, 5 ml preculture of *Chlorella* and 15 ml BG‐11 medium were loaded in 50 ml Falcon tubes and centrifuged at 4000 rpm at room temperature in a swing‐out rotor (Rotanta 460R; Hettich, Tuttlingen, Germany) for 15 min. The supernatant was separated from the pellet and 5 ml preculture of *Microcystis* was added to the supernatant to obtain mixtures of *Microcystis* with spent medium of *Chlorella* (treatment 4).

The pellets were harvested for disruption to obtain cellular lysate of *Chlorella*. For this purpose, pellets were transferred to 2 ml microcentrifuge tubes pre‐loaded with 0.3 g of 0.1 mm zirconia/silica acid‐washed beads, to which we added 1 ml of lysis buffer consisting of Tricine‐NaOH pH 7.4, 10 mM MgCl_2_, 5 mM NaCl, 10 mM NaK phosphate buffer pH 7.4 and 1 mg of the protease inhibitor phenylmethylsulfonyl fluoride (all final concentrations). Cell preparations were disrupted in a mini beadbeater‐8 (Biospec Products, Bartlesville, USA) during three alternating cycles of 1 min agitation and 2 min cooling. During the cooling phase, cells were kept at 4°C in a melting ice bath to avoid denaturation of enzymes. Subsequently, the supernatant was separated from the cellular debris and beads by centrifugation in a 4°C pre‐cooled Eppendorf Microfuge for 1 min at 12 000*g*. The supernatant served as the crude extract from which dilute cellular lysate was made by adding BG‐11 to a final volume of 5 ml. This cellular lysate was combined with 5 ml preculture of *Microcystis* and 15 ml BG‐11 medium to obtain mixtures of *Microcystis* with cellular lysate of *Chlorella* (treatment 5).

### Experiment 3: Density dependence of interspecific protection

In Experiment 3, we investigated whether the survival of *Microcystis* exposed to H_2_O_2_ depends on the population density of *Chlorella*. First, we measured H_2_O_2_ degradation by *Chlorella* monocultures at three different population densities: low *Chlorella* density (0.8 × 10^6^ cells ml^−1^), intermediate *Chlorella* density (2.7 × 10^6^ cells ml^−1^) and high *Chlorella* density (6.7 × 10^6^ cells ml^−1^). The *Chlorella* monocultures were treated with different H_2_O_2_ additions (10, 20 and 40 mg L^−1^), in triplicate. The H_2_O_2_ concentration was measured at *t* = 0, 1, 2, 3, 4 and 5 h after H_2_O_2_ addition.

Next, we investigated mixtures of *Microcystis* and *Chlorella*, consisting of 13.6 × 10^6^ cells ml^−1^ of *Microcystis* and the three above‐mentioned *Chlorella* densities, following the same procedures as in Experiment 2. These mixtures were exposed to six different H_2_O_2_ concentrations (0, 5, 10, 15, 20 and 40 mg L^−1^). After 5 days, population densities of *Microcystis* and *Chlorella* were counted.

### Experiment 4: H_2_O_2_ degradation by spent medium and cellular lysate

In Experiment 4 we investigated whether spent medium or cellular lysate of the organisms could degrade H_2_O_2_, e.g. by extracellular enzymes released in the spent medium or intracellular enzymes in the lysate. For this purpose, we prepared spent medium and cellular lysate of *Chlorella* monocultures and *Microcystis* monocultures, following the procedures described for Experiment 2. Population densities remaining in the spent medium were 0.8 × 10^6^ cells ml^−1^ for *Microcystis* and 0.2 × 10^6^ cells ml^−1^ for *Chlorella*, which is <10% of the population densities in the original samples. The cellular lysates did not contain intact cells, as confirmed by flow cytometry. At the start of the experiment, H_2_O_2_ was added to the incubation flasks at six different initial concentrations (0, 2.5, 5, 10, 15 and 20 mg L^−1^), and subsequent changes in H_2_O_2_ concentration were monitored.

### H_2_O_2_ analysis, cell counts and biovolumes

In all laboratory experiments, H_2_O_2_ concentrations were measured in triplicate using p‐nitrophenyl boronic acid as a reagent according to Lu *et al*. (2011). The H_2_O_2_ dependent formation of di‐nitrophenol was quantified by the absorbance at 405 nm measured with a Versamax microplate reader (Molecular Devices, Sunnyvale, CA, USA).

Biovolumes were quantified with a Casy 1 TTT cell counter (OLS OMNI Life Science, Bremen, Germany) with a 60 μm capillary.

A BD Accuri C6 flow cytometer (BD Biosciences, San Jose, USA) was used to distinguish *Microcystis* and *Chlorella* cells and count their population densities. The flow cytometer was equipped with a blue argon laser (488 nm) with green (533 nm, FL1), orange (585 nm, FL2) and red (670 nm, FL3) fluorescence detectors, a red diode laser (640 nm) with red fluorescence detector (675 nm, FL4), and photodiodes for forward scatter (FSC) and side scatter (SSC). A gating strategy was applied by plotting FSC versus log FL4 to distinguish cells from noise due to cellular debris. Subsequently, plots of FSC versus log FL3 were used to discriminate between the two species.

### Photosynthetic yield

The photosynthetic yield was used as a proxy for the vitality of phytoplankton and was determined with a portable Mini‐PAM‐2 fluorometer according to the manufacturer's instructions (Walz, Effeltrich, Germany). The 12‐well plates containing phytoplankton cultures were dark adapted for 10 min before the photosynthetic yield was measured with the sensor of the Mini‐PAM‐2 fluorometer mounted just above the wells. The photosynthetic yield *F*_v_/*F*_m_ (also known as the maximum quantum yield of PSII electron transport) was calculated as:

(1) $$F_{v}/F_{m}=\left(F_{m}–F_{0}\right)/F_{m},$$

where *F*_0_ is the minimum fluorescence and *F*_m_ is the maximum fluorescence in the dark following a saturating light pulse (Maxwell and Johnson, 2000).

### Lake treatments

To investigate how the presence of green algae affected the mitigation of harmful cyanobacterial blooms in lakes, we compared H_2_O_2_ treatments in two lakes in The Netherlands. The lake treatments followed the methods and procedures described in Matthijs *et al*. (2012). Lake Oosterduinse Meer (52° 16′ 55″ N, 4° 30′ 28″ E) had a surface area of 0.3 km^2^ and an average depth of 7 m. This lake was treated on 16 June 2016 with ~5 mg L^−1^ H_2_O_2_ to suppress the potentially toxin‐producing cyanobacterium *Aphanizomenon flos‐aquae*. In Lake Krabbeplas (51° 54′ 53″ N, 4° 18′ 10″ E), an isolated partition with a surface area of 0.03 km^2^ and an average depth of 1.75 m was treated on 28 June 2016 with ~4.6 mg L^−1^ H_2_O_2_ to suppress the potentially toxin‐producing cyanobacteria *Pseudanabaena limnetica*, *Planktothrix agardhii* and *Coelomoron pusillum*. Both lakes had been closed for recreation by the local water management due to public health risks. During and after the treatment, H_2_O_2_ concentrations in the lakes were measured at multiple time points using Quantofix indicator sticks (Macherey‐Nagel, Düren, Germany).

Phytoplankton was sampled from the top 30 cm of the lake, from 3 days prior up to 7 days after the H_2_O_2_ treatment in Lake Oosterduinse Meer, and from the day of treatment up to 9 days after the H_2_O_2_ treatment in Lake Krabbeplas. Phytoplankton samples were preserved with 0.4% Lugol's iodine solution and stored in the dark at 4°C until microscopic analysis. Phytoplankton was identified to species level when possible and counted with an inverted microscope (Zeiss IM35, Oberkochen, Germany) using a 1 ml counting chamber following the Utermöhl method (Utermöhl, 1958). Biovolumes of the phytoplankton were calculated from cell numbers and cellular geometry according to Hillebrand *et al*. (1999).

## Author Contributions

E.F.J.W., H.C.P.M., P.M.V. and J.H. designed the study. E.F.J.W. performed the lab experiments with technical support by H.C.P.M., J.M.S. and C.A.M.S. E.F.J.W., T.P. and P.M.V. performed the sampling and measurements during the lake treatments. M.J.v.H. identified and quantified the phytoplankton in samples of Lake Oosterduinse Meer. E.F.J.W. and J.H. wrote the manuscript, and all authors except our late colleague H.C.P.M. commented on the final version.

## Supporting information


**Fig. S1.** Minimum fluorescence (F_0_) and maximum fluorescence (*F*_m_) in monocultures of three species of freshwater cyanobacteria (*Microcystis*, *Anabaena* and *Planktothrix*) after addition of different H_2_O_2_ concentrations. Data show mean ± standard deviation (*n* = 3 per data point).
**Fig. S2.** Minimum fluorescence (F_0_) and maximum fluorescence (F_m_) in monocultures of six species of freshwater green algae (*Chlorella*, *Desmodesmus*, *Kirchneriella*, *Ankistrodesmus*, *Monoraphidium* and *Chlamydomonas*) after addition of different H_2_O_2_ concentrations. Data show mean ± standard deviation (*n* = 3 per data point).
**Fig. S3.** H_2_O_2_ degradation by the lysate and spent medium is slow in the absence of intact cells of *Chlorella* and *Microcystis*. Graphs show H_2_O_2_ degradation by the lysate and spent medium of both *Chlorella* and *Microcystis*, during the first 3 h after addition of different H_2_O_2_ concentrations.

**Table S1.** Phytoplankton community before and after H_2_O_2_ treatment of Lake Oosterduinse Meer on 16 June 2016. Samples were collected 3 days prior to H_2_O_2_ addition, and after 1, 2, 4, and 7 days. At the treatment day, two samples were taken: one sample one hour before the H_2_O_2_ addition started and another sample five hours after all H_2_O_2_ was added.
**Table S2.** Phytoplankton community before and after H_2_O_2_ treatment of Lake Krabbeplas on 28 June 2016. Samples were collected one hour before H_2_O_2_ addition started on the treatment day, and after 1, 2, 6, 7 and 9 days.
