# Supplementary material for: Interspecific protection against oxidative stress: green algae protect harmful cyanobacteria against hydrogen peroxide
Source: Environ Microbiol. 2021 Feb 21;23(5):2404–19. doi: 10.1111/1462-2920.15429 (PMC8248038; doi:10.1111/1462-2920.15429)
Supplement: Supplementary file 1 — Fig. S1. Minimum fluorescence (F0) and maximum fluorescence (F m) in monocultures of three species of freshwater cyanobacteria (Microcystis, Anabaena and Planktothrix) after addition of different H2O2 concentrations. Data show mean ± standard deviation (n = 3 per data point). Fig. S2. Minimum fluorescence (F0) and maximum fluorescence (Fm) in monocultures of six species of freshwater green algae (Chlorella, Desmodesmus, Kirchneriella, Ankistrodesmus, Monoraphidium and Chlamydomonas) after addition of different H2O2 concentrations. Data show mean ± standard deviation (n = 3 per data point). Fig. S3. H2O2 degradation by the lysate and spent medium is slow in the absence of intact cells of Chlorella and Microcystis. Graphs show H2O2 degradation by the lysate and spent medium of both Chlorella and Microcystis, during the first 3 h after addition of different H2O2 concentrations. [file EMI-23-2404-s002.pdf]

Supporting figures S1, S2 and S3 to:

Interspecific protection against oxidative stress: green algae protect harmful cyanobacteria against hydrogen peroxide

Erik F.J. Weenink[1], Hans C.P. Matthijs[1†], J. Merijn Schuurmans[1], Tim Piel[1], Maria J. van Herk[1], Corrien A.M. Sigon[1], Petra M. Visser[1] and Jef Huisman[1]

[1]Department of Freshwater and Marine Ecology, Institute for Biodiversity and Ecosystem Dynamics, University of Amsterdam, P.O. Box 94240, 1090 GE Amsterdam, The Netherlands.

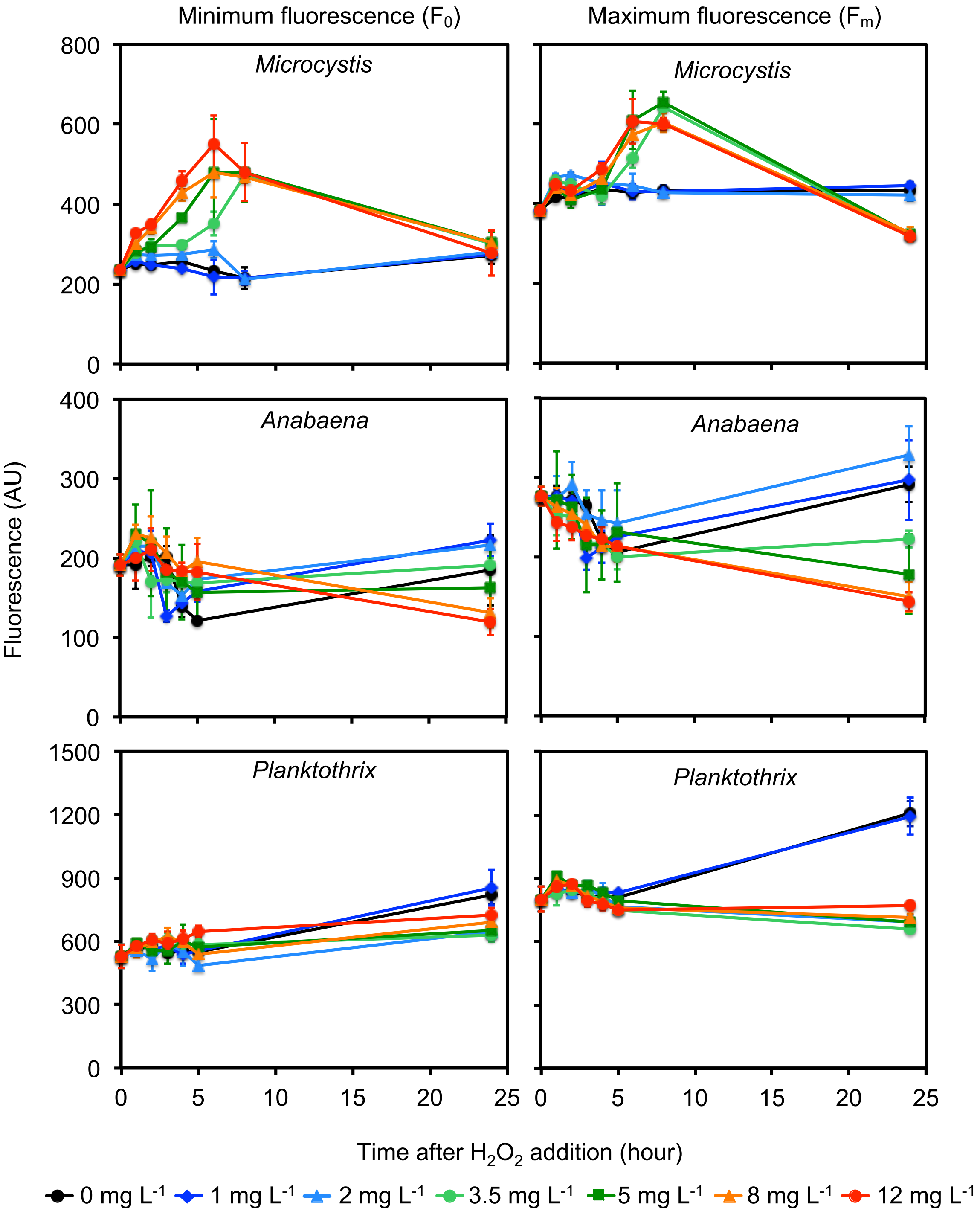

Fig. S1. Minimum fluorescence (F_0) and maximum fluorescence (F_m) in monocultures of three species of freshwater cyanobacteria: *Microcystis aeruginosa* strain PCC 7806, *Anabaena* PCC 7938 and *Planktothrix* PCC 7811, after addition of different H_2O_2 concentrations.

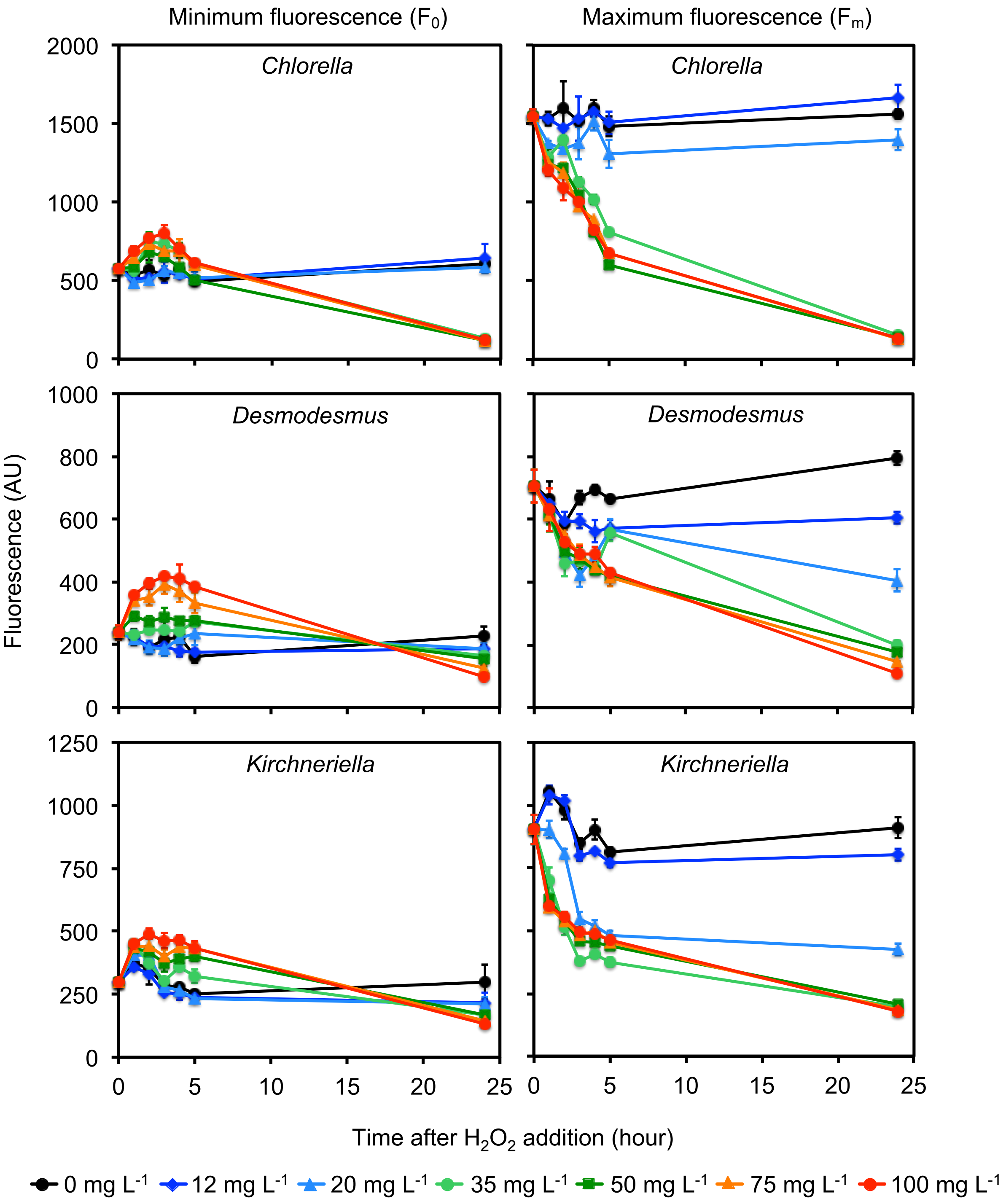

Fig. S2. Minimum Fluorescence (F_0) and maximum fluorescence (F_m) in monocultures of six species of fresh-water green algae: *Chlorella sorokiniana* SAG 211-8k, *Desmodesmus armatus* SAG 276-4e, *Kirchneriella contorta* SAG 11.81, *Ankistrodesmus falcatus* SAG 202-9, *Monoraphidium graffithii* SAG 202-13 and *Chlamydomonas reinhardtii* SAG 77.81, after addition of different H_2O_2 concentrations.

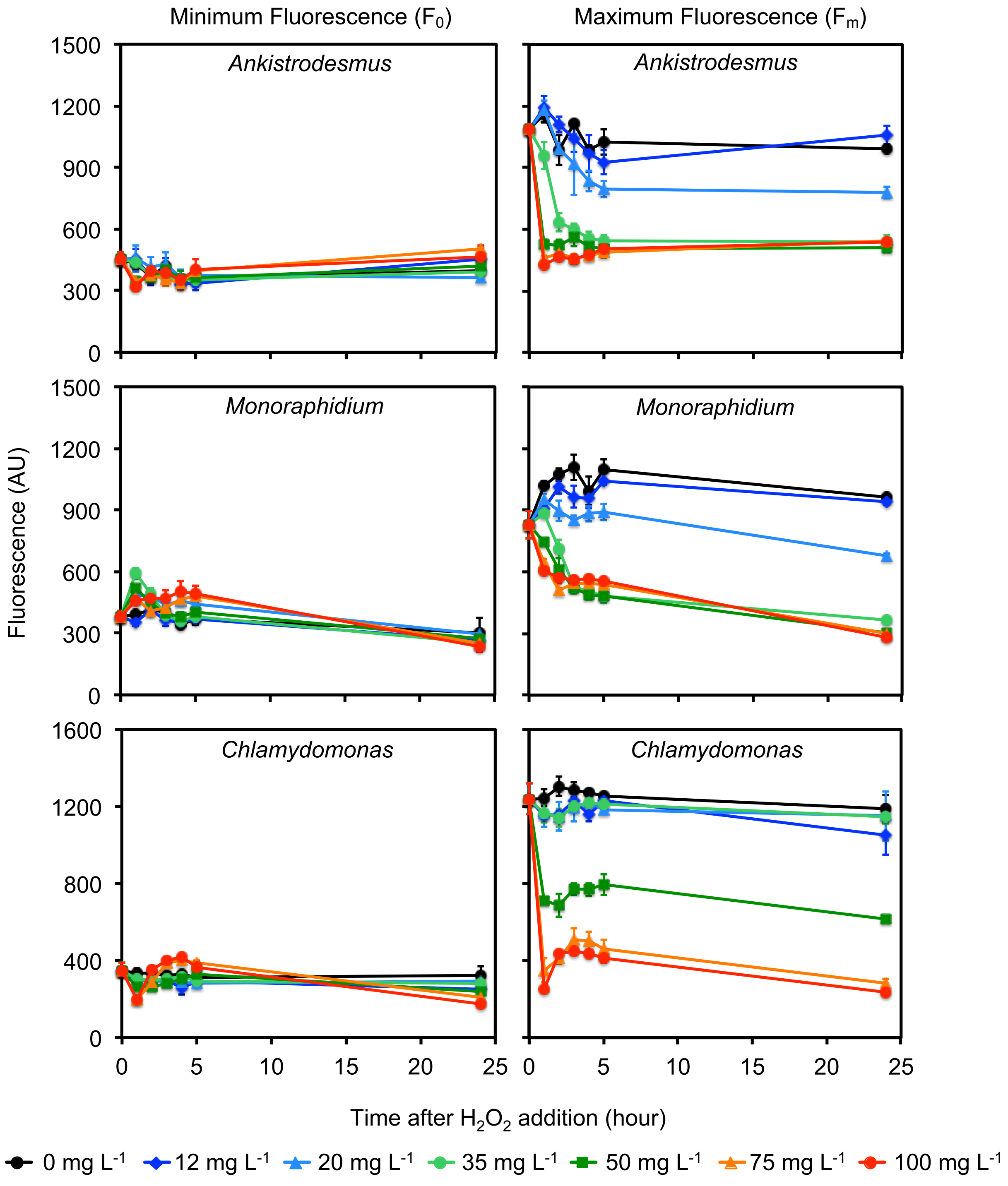

Fig. S2. (continued)

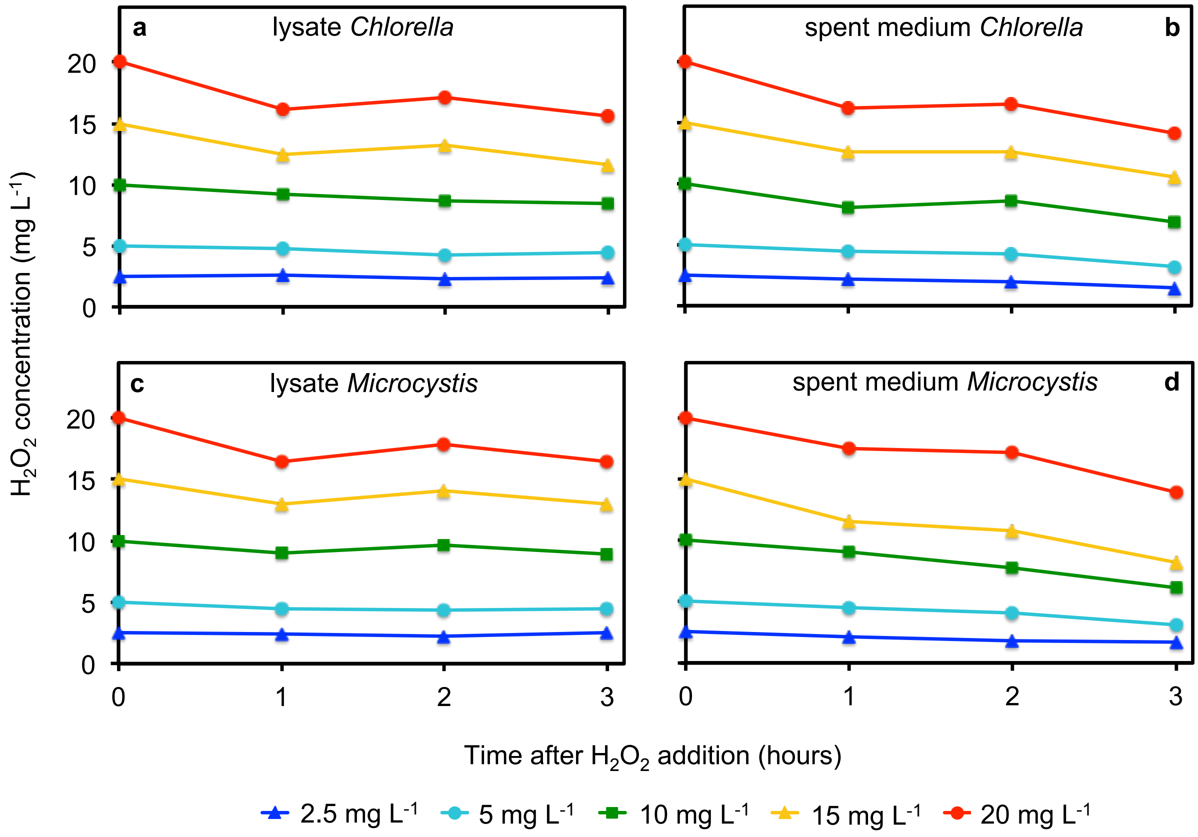

Fig. S3. H_2O_2 degradation by the lysate and spent medium is slow in the absence of intact cells of *Chlorella* and *Microcystis*. Graphs show H_2O_2 degradation by the lysate and spent medium of both *Chlorella* and *Microcystis*, during the first 3 hours after addition of different H_2O_2 concentrations.
